# Patients’ Non-Medical and Organizational Needs during Cancer Diagnosis and Treatment

**DOI:** 10.3390/ijerph17165841

**Published:** 2020-08-12

**Authors:** Karolina Osowiecka, Radoslaw Sroda, Arian Saied, Marek Szwiec, Sarah Mangold, Dominika Osuch, Sergiusz Nawrocki, Monika Rucinska

**Affiliations:** 1 Department of Psychology and Sociology of Health and Public Health, School of Public Health, University of Warmia and Mazury in Olsztyn, Al. Warszawska 30, 11-041 Olsztyn, Poland; 2 Department of Oncology, Collegium Medicum, University of Warmia and Mazury in Olsztyn, Al. Wojska Polskiego 37, 10-228 Olsztyn, Poland; sr.radoslaw@gmail.com (R.S.); arisai@wp.pl (A.S.); sergiusz.nawrocki@me.com (S.N.); m_rucinska@poczta.onet.pl (M.R.); 3 Department of Surgery and Oncology, Faculty of Medicine and Health Sciences, University of Zielona Gora, Ul. Zyty 28, 65-046 Zielona Gora, Poland; szwiec72@gmail.com; 4 Department of Oncology and Radiotherapy, Medical University of Silesia in Katowice, Ul. Ceglana 35, 40-515 Katowice, Poland; sarah.mangold@wp.pl (S.M.); dominika.osuch@onet.pl (D.O.)

**Keywords:** non-medical needs, cancer, psychological support, social support, oncological system

## Abstract

The aim of this cross-sectional study was to determine non-medical and organizational needs among cancer patients during diagnosis and treatment. The study included 384 cancer patients treated in five oncological centers in Poland. A questionnaire designed for the study was used. Most of the patients received psychological support from their partner/family/friends (88%), to a lesser extent from a psychologist (21%) and priests (4%). Forty-three percent of patients received social support from their partner/family/friends and only 7% of respondents received support from a social worker. Most patients stated they would like to have a professional who would help them with their non-medical problems during the diagnostic process and cancer treatment. The youth, with a higher education level who were professionally active and living in cities seemed to be more aware of their needs. Improvements to the oncological system in Poland should focus on expanding patient access to professional support of non-medical needs.

## 1. Introduction 

Cancer is a leading cause of death after cardiovascular diseases. The World Health Organization estimated the number of new cancer cases worldwide in 2018 to be over 18 million [1]. The crude rate and age-standardized rate worldwide was 236.9 and 197.9 per 100,000, respectively. In Poland, in 2017, the crude rate and age-standardized rate per 100,000 was 429.11 and 230.14, respectively [2]. The earlier diagnoses and improvements to cancer therapies provide increasing survival rates. Cancer is a very serious medical problem, requiring complex and multidisciplinary treatment, but is also a very stressful event with significant psychosocial implications related to physical, emotional, spiritual, and interpersonal dimensions. There are many spheres of life affected by cancer diagnosis and therapy. Patients with cancer often have physical, emotional, interpersonal, and informational difficulties that in one way or another prevent them from getting the optimal treatment for their disease. There is lots of evidence that people with cancer suffer from psychosocial distress, including many emotional, cognitive, social, and functional problems, which have been documented in many studies [3]. Their families are also affected and experience emotional distress, a shifting of roles, financial burdens, caregiver stress, and the fear of losing their loved ones [4]. Psychological consequences of cancer and cancer treatment at the physical level (in body images like amputations, stomas, and hair loss) were also noted [5]. Hope and social support have a positive and statistically significant impact on the resilience of cancer patients [6]. Some authors investigated that emotional and social support can help patients learn to cope with psychological stress, reduce the level of depression, anxiety, and disease- and treatment-related symptoms among patients [7]. The identification of cancer patients’ needs may improve care and help achieve cancer patients’ satisfaction [8,9]. It should pay more attention to non-medical areas of patients’ life including emotional consequences, supportive care needs, and quality of life of cancer patients and their families. Psychologists, social workers, dieticians, and physiotherapists ect., may support patients in non-medical areas. There is a question whether patients are sufficiently informed about the possibility of, and their satisfaction with, support. Sometimes it may be difficult for patients to express their own needs to medical staff [10].

The purpose of this study was to determine the non-medical (psychological, social, and spiritual needs not related directly to medical care) and organizational needs (access to diagnostics, treatment, and social services) among cancer patients, and assess how these needs are being fulfilled. The analysis included cancer patients’ problems with professional activity during the cancer diagnosis and treatment, with getting hold of social services, and with access to diagnostic or therapeutic procedures. Sources of psychological and social support were analyzed. The relationship between non-medical needs and demographic factors were investigated.

## 2. Materials and Methods

The study was conducted on a group of 384 patients who were treated for cancer between April 2018 and March 2019 in five oncological centers in Poland (1 in north-eastern, 2 in central, 1 in southern, and 1 in south-western regions, respectively: Hospital of the Ministry of Internal Affairs with Warmia and Mazury Oncology Center in Olsztyn; Military Institute of Medicine in Warsaw; The Maria Sklodowska-Curie Institute—Oncology Center in Warsaw; University Center for Ophthalmology and Oncology, Medical University of Silesia in Katowice; University Hospital in Zielona Gora). The cancer centers invited to participate in this study were public resources. 

Patients: The inclusion criteria were: cancer diagnosis, age ≥ 18 years old, actively being treated for cancer, and with a signed consent to participate in the study. The participants were treated as in- or outpatient at radiotherapy and/or chemotherapy departments. Interviewers (medical students, nurses) administered the questionnaire during the interview with patients.

Questionnaire: A questionnaire (Table S1) was designed specifically for this study. At the beginning, the questionnaire had been validated by a group of 20 patients. The validation procedure included a questionnaire that was carried out twice in a two-week interval on the same group of patients. The measure of compliance was calculated using Cohen’s Kappa coefficient. The questionnaire consisted of 9 main closed-ended quantitative questions (with the additional option of “other” for expanded/not included answers) and 6 questions concerning demographic data. The data on the tumor localization were supplemented with medical records or hospital databases.

The study protocol was approved by the Ethics Committee of University of Warmia and Mazury in Olsztyn (2/2018). Participation in the study was voluntary. All study participants gave their consent and signed it.

### Statistical Analysis

The study comprised of a cross-sectional analysis. The validation of the questionnaire was carried out using Cohen’s Kappa. The distributions of continuous variables were compared with the theoretical normal distribution using the Shapiro–Wilk test. The differences between the subgroups were analyzed with either the Mann–Whitney (for 2 subgroups) or the Kruskal–Wallis test, and the Dunn’s test post hoc (for >2 subgroups). A comparison of the proportion in subgroups was tested using the chi-square test. A *p*-value of < 0.05 was considered to be significant. The analysis was conducted using STATISTICA software (version 13.3) (StatSoft, Krakow, Poland) and SPSS Statistics 23.0 (IBM, Armonk, NY, USA). 

## 3. Results

The study was carried out on a group of 384 patients diagnosed with various localizations of cancer treated in five oncological centers in Poland. The analysis included 203 women and 181 men aged 18–93 years (median age 65 years). The majority of the patients had graduated from secondary school (58%), were pensioners (56%), married (69.8%), and lived in cities (74.7%) (Table 1). 

### 3.1. Problems with Access to Diagnostic and Therapeutic Procedures

One third of the patients (32%) paid for some of the diagnostic examinations. Patients with higher education, more often than patients with lower education levels, used private services (respectively 56% and 25%; *p* < 0.001). One-fifth of the patients claimed difficulties during the diagnostic process. Three patients (4%) received the refusal of a referral for examination, 49 patients (63%) waited a long time for a test/visit, 19 patients (24%) waited a long time to receive treatment and 15 patients (19%) declared other problems. Patients with a higher education showed significantly more frequent problems (*p* = 0.03). Most of the patients (91%) experienced no inconvenience during the cancer treatment. Women (*p* = 0.04) and younger patients (*p* = 0.01) with a higher education (*p* < 0.001) who were professionally active (*p* = 0.04) and were living in cities (*p* = 0.018) in the southern region of Poland (*p* = 0.008) reported these problems more often (Table 2).

### 3.2. Psychological Support

**Figure 1:**
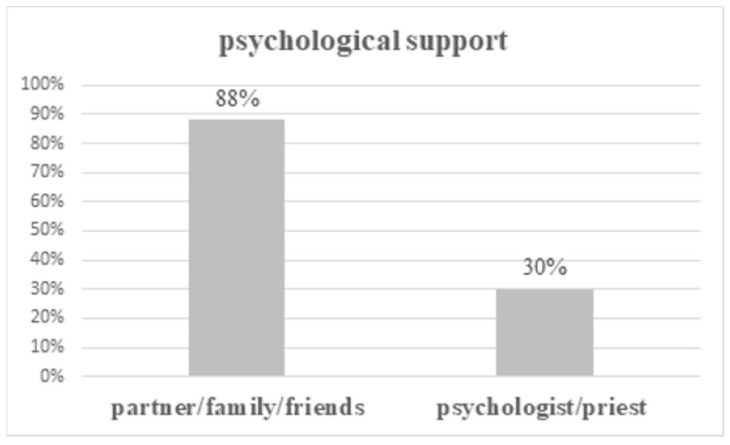
Psychological support for cancer patients (some patients received support from more than one helper).

Seventy-six percent of cancer patients needed psychological support, the majority received this kind of support from a partner and/or family (251/290 patients; 65%) and friends (86/290 patients; 22%), fewer patients received help from a psychologist (81/290 patients; 21%) or a priest (17/290 patients; 4%) (Figure 1). 

### 3.3. Social Support

**Figure 2:**
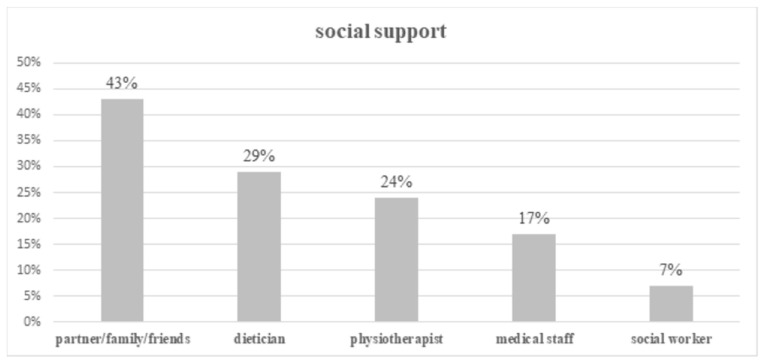
Social support for cancer patients (some patients received support from more than one helper).

Fifty percent of patients required social support. Again, the main source of support was from a partner and/or family (78/192 patients; 41%) and friends (22/192 patients; 11%), only 7% (13/192 patients) received professional help from a social worker. The help from a dietician was given to 29% of cancer patients (56/192 patients). Twenty-four percent of patients (47/192 patients) received support from a physiotherapist and 17% of patients (32/192 patients) received support from other medical staff (Figure 2).

It turned out that married patients could significantly more often count on psychological support than single or widow/er patients (*p* = 0.03). The region of Poland where patients live also influenced their ability to gain psychological and social help (*p* < 0.05). Tumor localization was correlated with social support (*p* = 0.02). Rectum cancer patients more often could count on dietician, physiotherapist, and social worker help. The study showed that some patients did not even know that they could expect support and assistance in their non-medical needs (16% of patients in cases of psychological support and about 25% of patients in cases of social support) (Table 3).

### 3.4. Professional Activity

One hundred and ten individuals’ cancer diagnosis influenced their professional activity, because of the need to use a long health leave (66/110 patients; 60%), a lost job (19/110 patients; 17%), or the need for an early retirement (26/110 patients; 24%).

### 3.5. Social Service

Two thirds of patients (67%) declared no problems with getting social service (financial support, getting a pension, orthopedic equipment, etc.) The significant differences in getting social services were observed between various regions of Poland (*p* < 0.001) (Table 4).

Most cancer patients (79%) declared that they would need a professional who could help and support them with their non-medical problems accompanying cancer diagnosis and treatment.

## 4. Discussion

Cancer diagnosis and treatment affects patients’ lives, brings changes in patients’ daily activities, work, relationships, and family roles. It is associated with a high level of patient psychological stress, but this problem seems to be still marginalized and underestimated [11]. A supportive system directed at overcoming patient’s stressful experiences should include information, emotional help, and material support (services that help practical problems) [12].

In our study, patients most frequently received psychological support from their partner or/and family or/and friends (88%) in comparison with a psychologist or/and a priest (30%). Psychological help was significantly more frequent in married than in single or widowed patients, probably most of this came from partners. The study conducted by Slevin et al. [13] confirmed that family (73%) and friends (52%) were the sources of emotional support which patients would most likely take advantage of next to senior doctors (73%) and consultants (63%), but not psychologists and social workers. In breast cancer patients the most important supporters were partners (94.3%), a close relative (12.0%), and friends (5.4%) [14]. Unfortunately, the participation of professionals is minimal. This was also confirmed by our results: only 29% of patients claimed that they received help from dieticians, 24% of patients from physiotherapists, and 17% of patients from other medical staff. Bonacchi et al. [15] determined unmet needs among inpatients and outpatients: patients indicated the need to speak with a psychologist (22.5%), with a spiritual assistant (15.4%), with people who have had the same experience (40.6%), and had a need to have more economic-insurance information in relation to their illness (40.1%), and had a need for economic help (16.9%).

Some authors showed a positive association between perceived social support and psychological adjustment following cancer treatment [16]. Söllner et al. [17] estimated that high social support combined either with active coping or with the stoicism of early-stage melanoma patients was associated with a good adjustment to cancer, whereas a low perceived support in the subjects of living alone or in patients exhibiting depressive coping behavior was associated with poor adjustment. The authors indicated a relationship between psychological disorders and a higher risk of recurrence and decreased overall survival because of reduced response to chemotherapy among depressed breast cancer patients [18].

Slevin et al. [13] showed a significant difference based on the age group of patients: patients opting for health professionals were in the older age group, while younger patients were more likely to use family, friends and other patients. In our study we did not determine any significant difference in getting psychological help, since only patients from the south-western region had psychosocial support regularly and patients with diagnosed rectum cancer frequently counted on social support.

Forsythe et al. [19] indicated that only 40% of survivors reported discussing with their clinicians about how cancer may have affected their emotions or relationships and that more than 90% of survivors did not use professional support because of a lack of knowledge or an unavailability of services. It is important to monitor the supportive care needs in patients, especially in those who report a fear of recurrence and anxiety [20]. It turns out that the risk of psychological morbidity is high in patients with an active or a recurrent disease [21]. In our study, some patients also did not realize that they could receive help with their non-medical needs. Cancer care professionals tend to mistake clinical depression with normal sadness and believe that it is normal to feel sad or anxious because of cancer. About 30–40% of cancer patients are not identified by their clinician and therefore they are not referred to the psychiatric or psycho-oncology services [22]. The informational needs were regarded to be as follows: concerning diagnosis (40%), information about future conditions (61%), regarding a better dialogue with clinicians (45%), and economic-insurance information (40%); and the need for better services at the hospital like bathrooms, meals, and cleaning (59%) [23]. Patients wanted to know more about their own diagnosis and future conditions [10,23,24,25,26]. However, the open communication in the doctor–patient relationship is important and some studies noticed the necessity for clinicians to help patients to understand medical information [24,27].

Most patients did not report any difficulties in diagnostic or the therapeutic system. Among patients who declared difficulties during the diagnostic process, 4% of patients received a refusal of a referral for examination, 63% of patients waited a long time for a test/visit, 24% of patients waited a long time to receive treatment and 19% of patients declared other problems. The previously published results showed the waiting time for diagnosis and treatment after cancer suspicion in Poland is too long (about 11 weeks) [28,29]. The presented study showed that patients with a higher education are more aware of the delay in the diagnosis. One third of the patients used private services during the diagnostic process and more than half of the patients with a higher education paid for healthcare services in comparison with the patients with lower education levels. Patients who declared more difficulties during treatment were as follows: women, younger patients, those that were professionally active, those with a higher education, and those living in cities and in the southern regions of Poland. We earlier published [29] that the patients who were likely to wait significantly longer for their diagnosis from cancer suspicion were pensioners, and also that the waiting time was correlated with region. The factors that significantly influenced the shorter time from diagnosis to treatment were as follows: higher education, place of residence in larger cities, and professionally active. In our current research we can confirm that a large number of patients pay for medical services (almost 1/3) despite the fact that in Poland all citizens are guaranteed free medical care. That group of patients is aware of how the impact of a difficult access to health service affects cancer, treatment outcomes, and survival, which has also been discovered by several other authors [30,31,32,33,34,35].

Our study proved that cancer diagnosis influenced professional activity, due to the need to use a long health leave (60%), a lost job (17%), and the need for early retirement (24%). Loss of work decreases household income, resulting in a reduced quality of daily life [36]. Levels of work incapacity among head and neck cancer patients vary from 34 to 52% [36,37,38]. The type of treatment appears to have an impact on the resumption of professional activity. Only 15% of patients undergoing total laryngectomy and 50% of those undergoing supraglottic laryngectomy go back to work after surgery [39].

In our study, most patients (about 80%) considered it necessary for a special person to provide for them in an oncological system with informative, emotional, and social support and to help them to find a solution to various problems. In Poland there is *a coordinator of cancer treatment*, who has been established since 2015, but the main role of this person focuses on informative and administrative functions. Half of the people employed as *a coordinator of cancer treatment* have other main duties and most of them have never been trained. The authors underlined the importance of skills, knowledge, and experience of any person who helps navigate patients in the system [40,41]. In Israel, the role of coordinator has been given to a special nurse who supports cancer patients emotionally and in moving in healthcare [41]. Swanson et al. [42] found a statistically significant lower stress in cancer patients from Saint Elizabeth Regional Medical Center in Lincoln when they received help from the coordinating nurse. In the randomized control trial conducted by Fiscella et al., in some cancer centers in USA [43], the introduction of the coordination program had had a positive influence on increasing the satisfaction of health care in patients with breast and colon cancer. Patients under special cancer coordination received proper psychological support and help in informative needs and in solving problems [44,45].

### Study Limitations

The study was limited by including patients from only five oncological centers. The study was prospective but not all patients under treatment during the study period were included. The questionnaire did not cover all patients’ non-medical needs. The study was limited by a lack of qualitative analysis, which could provide valuable conclusions.

## 5. Conclusions

Social and psychological support plays an important role in a cancer patient’s wellbeing during the diagnostic process and therapy. In Poland, most often the psychological and social support was given by partners and family rather than by professional specialists. Some patients claimed that they did not know about the possibility of using psychological and social support in the system. Younger people with a higher education, who were professionally active, and living in cities seem to be more aware of their needs. One third of cancer patients in Poland used private services during diagnosis. It would be important to find an opportunity to improve the oncological system in Poland to limit the necessity to pay more for services by patients (e.g, quick diagnosis) and to improve access to professional support for non-medical needs.

## Figures and Tables

**Table 1:** Characteristic of patients.

		*n*	%
		384	100.0
Age (years)	range 18–93; average 63 ± 11.9; median 65		
Gender			
	women	203	52.9
	men	181	47.1
Education			
	primary	75	19.5
	secondary	223	58.1
	high	79	20.6
	no data	7	1.8
Professional activity			
	student	4	1.1
	employed	133	34.6
	unemployed	27	7.0
	pensioner	215	56.0
	no data	5	1.3
Marital status			
	married	268	69.8
	single	58	15.1
	widow/er	53	13.8
	no data	5	1.3
Place of residence			
	city	287	74.7
	village	96	25.0
	no data	1	0.3
Region of Poland			
	north-east	56	14.6
	centrum	96	25.0
	south	103	26.8
	south-west	129	33.6
Cancer			
	esophagus or stomach	41	10.6
	rectum	71	18.5
	breast	74	19.3
	gynecological	30	7.8
	head and neck	39	10.2
	prostate	9	2.3
	brain	14	3.6
	melanoma	1	0.3
	lung	72	18.8
	urinary system	9	2.3
	others	11	2.9
	no data	13	3.4

**Table 2:** Organizational problems of the care system.

	Difficulties during Cancer Diagnosis					Difficulties during Cancer Treatment					Using Private Services during the Diagnostic Process				
	**yes**		**no**			**yes**		**no**			**yes**		**no**		
	*n*	%	*n*	%	*p*	*n*	%	no	%	*p*	*n*	%	*n*	%	*p*
	78	20	302	79		36	9	345	90		124	32	259	68	
Age (years)	range 25–85; average 62.5 ± 13.2; median 65		range 18–93; average 62.9 ± 11.6; median 64		0.93	range 26–85; average 58.3 ± 14.1; median 58.5		range 16–93; average 63.4 ± 11.6; median 65		0.01	range 23–85; average 61.9 ± 12.3; median 65		range 18–93; average 63.4 ± 11.7; median 64		0.39
Gender															
women	38	49	162	54	0.44	25	69	178	52	0.04	72	58	131	51	0.17
men	40	51	140	46		11	31	167	48		52	42	128	49	
Education															
primary	11	14	63	21	0.03	0	0	74	21	<0.001	17	14	58	22	<0.001
secondary	42	54	180	59		20	55	201	58		59	48	163	63	
high	24	31	54	18		15	42	64	19		44	35	35	14	
no data	1	1	5	2		1	3	6	2		4	3	3	1	
Professional activity															
student	0	0	4	1.5	0.25	0	0	4	1	0.04	1	1	3	1	0.33
employed	33	42	100	33		20	55	113	33		48	39	85	33	
unemployed	3	4	24	8		2	6	24	7		5	4	22	8	
pensioner	41	53	170	56		13	36	200	58		67	54	147	57	
no data	1	1	4	1.5		1	3	4	1		3	2	2	1	
Marital status															
married	53	68	212	70	0.75	27	75	240	70	0.61	92	74	175	68	0.15
single	14	18	44	15		5	14	52	15		18	15	40	15	
widow/er	10	13	42	14		3	8	49	14		11	9	42	16	
no data	1	1	4	1		1	3	4	1		3	2	2	1	
Place of residence															
city	63	81	221	73	0.12	32	89	253	73	0.018	95	76	191	74	0.46
village	14	18	81	27		3	8	92	27		28	23	68	26	
no data	1	1	0	0		1	3	0	0		1	1	0	0	
Region of Poland															
north-east	12	15	43	14	0.66	6	17	50	14.5	0.008	20	16	36	14	0.23
center	17	22	79	26		9	25	87	25		30	24	66	25	
south	25	32	78	26		17	47	86	25		40	32.5	63	24	
south-west	24	31	102	34		4	11	122	35.5		34	27.5	94	37	
Cancer															
esophagus or stomach	13	17	28	9	0.21	6	17	35	10.5	0.58	19	15	22	8.5	0.02
rectum	17	22	53	17.5		2	6	67	19		16	13	54	21	
breast	13	17	61	20		7	20	67	19		31	25	43	17	
gynecological	2	3	27	9		4	11	26	8		12	10	18	7	
head and neck	9	12	30	10		4	11	34	10		10	8	29	11	
prostate	4	5	5	2		1	3	8	2.5		5	4	4	2	
brain	2	3	12	4		3	9	11	3		5	4	9	3	
melanoma	0	0	1	0.5		0	0	1	0.5		0	0	1	0.5	
lung	11	14	59	19.5		5	14	67	19		14	11	58	22	
urinary system	3	4	6	2		1	3	8	2.5		2	2	7	3	
others	3	4	8	2.5		1	3	10	3		5	4	6	2	
no data	1	1	12	4		1	3	11	3		5	4	8	3	

**Table 3:** Psychological and social support of patients.

	Psychological Support							Social Support						
	yes		no		Do not Need to Use or Do not Know about the Possibility			yes		no		Do not Need to Use or Do not Know about the Possibility		
	*n*	%	*n*	%	*n*	%	*p*	*n*	%	*n*	%	*n*	%	*p*
	290	76.3	29	7.6	61	16.1		192	50.7	93	24.5	94	24.8	
Age (years)	range 16–93; average 62.3 ± 11.9; median 64		range 35–87; average 63.8 ± 10.9; median 65		range 33–85; average 65.5 ± 12.5; median 67		0.14	range 25–87; average 63.1 ± 10.8; median 65		range 23–93; average 62.6 ± 12.3; median 63		range 16–84; average 62.6 ± 13.6; median 65		0.73
Gender														
women	152	52	16	55	33	54	0.94	98	51	52	56	51	54	0.71
men	138	48	13	45	28	46		94	49	41	44	43	46	
Education														
primary	53	18	7	24	13	21	0.91	37	19	14	15	21	22.5	0.51
secondary	171	59	17	59	34	56		109	57	62	67	51	54	
high	59	20.5	5	17	14	23		43	22	17	18	18	19	
no data	7	2.5	0	0	0	0		3	2	0	0	4	4.5	
Professional activity														
student	4	1.5	0	0	0	0	0.84	1	0.5	0	0	3	3	0.28
employed	102	35	9	31	21	34		69	36	36	39	27	29	
unemployed	22	7.5	1	3	4	7		13	7	6	6	8	8.5	
pensioner	157	54	19	66	36	59		108	56	51	55	52	55	
no data	5	2	0	0	0	0		1	0.5	0	0	4	4.5	
Marital status														
married	209	72	15	51.5	41	67	0.03	138	72	62	67	64	68	0.25
single	44	15	6	20.5	7	12		33	17	13	14	11	11.5	
widow/er	32	11	8	28.0	13	21		20	10.5	18	19	15	16	
no data	5	2	0	0	0	0		1	0.5	0	0	4	4.5	
Place of residence														
city	218	75	18	62	48	79	0.22	139	72.5	69	74	75	80	0.43
village	71	24.5	11	38	13	21		52	27	24	26	19	20	
no data	1	0.5	0	0	0	0		1	0.5	0	0	0	0	
Region of Poland														
north-east	49	17	3	10	4	7	0.001	31	16	15	16	10	10.5	<0.001
center	59	20	8	28	28	46		42	22	22	24	31	33	
south	82	28.5	11	38	10	16		52	27	38	41	13	14	
south-west	100	34.5	7	24	19	31		67	35	18	19	40	42.5	
Cancer														
esophagus or stomach	34	11.5	1	3.5	6	9.5	0.21	28	14.5	10	11	3	3	0.02
rectum	52	18	7	24	11	18		36	19	12	13	22	23.5	
breast	48	16.5	6	21	19	31		27	14	20	21.5	26	28	
gynecological	27	9	0	0	2	3		20	10.5	6	6.5	3	3	
head and neck	31	11	5	17	3	5		22	11.5	10	11	7	7.5	
prostate	5	2	0	0	4	6.5		7	3.5	0	0	2	2	
brain	11	4	2	7	1	2		6	3	5	5.5	3	3	
melanoma	1	0.5	0	0	0	0		0	0	1	1	0	0	
lung	56	19	5	17	11	18		30	16	21	22.5	21	22.5	
urinary system	7	2.5	0	0	1	2		5	2.5	1	1	1	1	
others	9	3	1	3.5	1	2		5	2.5	4	4	2	2	
no data	9	3	2	7	2	3		6	3	3	3	4	4.5	

**Table 4:** Problems with getting social service according to various factors.

	Problems with Getting Social Service						
	yes		no		Do not Need to Use or Do not Know about Possibility		
	*n*	%	*n*	%	*n*	%	*p*
	22	5.8	255	66.9	104	27.3	
Age (years)	range 49–85; average 64.2 ± 10.1; median 62		range 23–93; average 62.5 ± 11.8; median 65		range 16–87; average 63.7 ± 12.5; median 65		0.62
Gender							
women	13	59	142	56	46	44	0.12
men	9	41	113	44	58	56	
Education							
primary	6	27	48	19	21	20	0.89
secondary	11	50	150	59	60	58	
high	4	18	52	20	22	21	
no data	1	5	5	2	1	1	
Professional activity							
student	0	0	2	1	2	2	0.28
employed	8	36	93	36	32	31	
unemployed	4	18	17	7	6	6	
pensioner	9	41	140	55	63	60	
no data	1	5	3	1	1	1	
Marital status							
married	15	67	180	71	71	68	0.92
single	3	14	40	16	15	14.5	
widow/er	3	14	32	12	17	16.5	
no data	1	5	3	1	1	1	
Place of residence							
city	19	86	184	72	81	78	0.12
village	2	9	71	28	23	22	
no data	1	5	0	0	0	0	
Region of Poland							
north-east	4	18	48	19	3	3	<0.001
center	4	18	54	21	38	37	
south	11	50	71	28	21	20	
south-west	3	14	82	32	42	40	
Cancer							
esophagus or stomach	3	13.5	26	10	12	11.5	0.27
rectum	5	22.5	42	16.5	23	22	
breast	2	9	56	22	16	15	
gynecological	1	4.7	20	8	8	8	
head and neck	1	4.7	29	11	9	8.5	
prostate	0	0	2	1	7	7	
brain	1	4.7	8	3	5	5	
melanoma	0	0	1	0.5	0	0	
lung	5	22.5	49	19	17	16	
urinary system	1	4.7	5	2	3	3	
others	2	9	7	3	2	2	
no data	1	4.7	10	4	2	2	

## Supplementary Materials

The following are available online at https://www.mdpi.com/1660-4601/17/16/5841/s1, Table S1: Questionnaire.
