# Supplementary material for: Patients’ Non-Medical and Organizational Needs during Cancer Diagnosis and Treatment
Source: Int J Environ Res Public Health. 2020 Aug 12;17(16):5841. doi: 10.3390/ijerph17165841 (PMC7459913; doi:10.3390/ijerph17165841)
Supplement: Supplementary file 1 [file ijerph-17-05841-s001.pdf]

Supplementary Materials:

Table S1 Questionnaire

1. Did cancer diagnosis influence on your professional activity?

- yes, how?
  - the need to use a long health leave
  - lost a job
  - the need for early retirement
  - other:
- no

2. Did you have any problems with getting social service (financial support, getting pension, orthopedic equipment etc.)?

- yes
- no
- do not need to use
- do not know about possibility

3. Did you have any difficulties during the diagnostic process with access to diagnostic procedures?

- yes, which?
  - refusal of a referral for examination/visit in specialist
  - long waiting time for diagnostic examinations/visit in specialist
  - long waiting time for getting oncological treatment
  - other:
- no

4. Did you have any inconveniences during the cancer treatment with access to therapeutic procedures?

- yes, which?
- no

5. Did you get diagnostic and cancer treatment card (possibility of access to quick oncological diagnostics and treatment)?

- yes
- no
- do not know

6. Did you use a private service during diagnostic process?

☐ yes, why?

☐ no

7. Could you count on, and have you received, psychological support during the cancer diagnosis and treatment?

☐ yes, from:
  - partner
  - family
  - friends
  - psychologist
  - priest
  - other:

☐ no

☐ do not need to use

☐ do not know about possibility

8. Could you count on, and have you received, social support during the cancer diagnosis and treatment?

☐ yes, from:
  - partner
  - family
  - friends
  - dietician
  - physiotherapist
  - social worker
  - other:

☐ no

☐ do not need to use

☐ do not know about possibility

9. Do you think that presence of a professional person in the cancer care system who would help and support cancer patients with the non-medical problems associated with cancer diagnosis and treatment is:

☐ necessary

- ☐ unnecessary
- ☐ I have no opinion

Demographics

10. Gender:
- ☐ women
- ☐ men

11. Age:

12. Education:
- ☐ primary
- ☐ secondary
- ☐ high

13. Place of residence
- ☐ city
- ☐ village

14. Professional activity before cancer diagnosis
- ☐ student
- ☐ employed
- ☐ unemployed
- ☐ pensioner

15. Marital status
- ☐ married
- ☐ single
- ☐ widow/er
